# Forces to pierce cuticle of tarsi and material properties determined by nanoindentation: The Achilles’ heel of bed bugs

**DOI:** 10.1242/bio.028381

**Published:** 2017-09-21

**Authors:** Jorge Bustamante, Jason F. Panzarino, Timothy J. Rupert, Catherine Loudon

**Affiliations:** 1 Department of Ecology and Evolutionary Biology, University of California at Irvine, Irvine, CA 92697, USA; 2 Department of Mechanical and Aerospace Engineering, University of California at Irvine, Irvine, CA 92697, USA

**Keywords:** Creep, Reduced elastic modulus, Insect, *Cimex lectularius*

## Abstract

The mechanical properties of bed bug (*Cimex lectularius* L.) tarsi and pretarsi were investigated in order to evaluate their vulnerability to piercing by plant trichomes (sharp microscopic hairs). Nanoindentation was used to measure the force required to insert a sharp probe into the cuticle of these different regions, as well as to determine creep and reduced elastic moduli for the cuticle. Scanning electron microscopy was used to visualize the indents that had been generated by nanoindentation. The force required to insert a cube corner nanoindenter probe into the cuticle was determined for a range of displacements (1 to 9 μm) and strain rates (0.003 to 0.5 s^−1^). Greater force was required to insert this sharp probe at greater depth or at faster strain rates. A specific region of the pretarsus (membrane with microtrichia) more frequently pierced by trichomes during bed bug locomotion required approximately 20-30% less force, exhibited more creep, and had a lower reduced elastic modulus for the first micron of indentation compared to the other regions; although this pattern was not consistent for greater displacements. These mechanical attributes, which will facilitate the initial stage of puncture in addition to the presence of natural infoldings in the cuticle of this area, may make that area of the pretarsus particularly vulnerable to piercing. This information will help inform development of physical methods for control of insect pests such as bed bugs.

## Abstract (precis)

**Summary:** Mechanical properties were measured for bed bug cuticle of the tarsi to evaluate physical plant defenses and to inform the development of novel biomimetic physical insecticides.

## INTRODUCTION

**Fig. 1.**
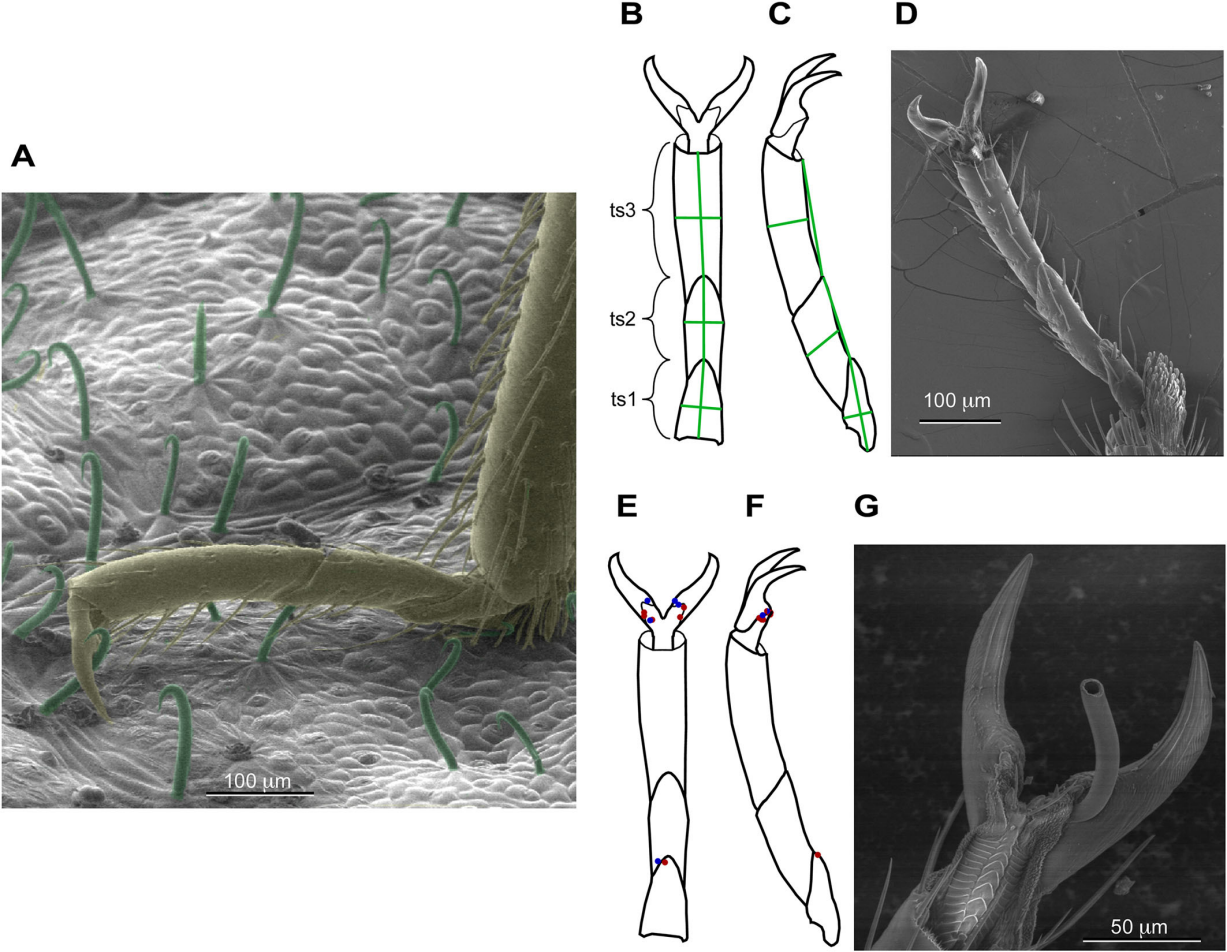
**Anatomy of tarsi and piercing locations.** (A) Scanning electron micrograph of the distal portion of a bed bug leg (brown) standing on a leaf from a bean plant (trichomes are green). The tibia of the bed bug leg is near the right margin, and the tarsus is roughly parallel to the leaf surface and terminates in two pretarsal claws. (B) The three tarsal subsegments (ts1, ts2, and ts3) are numbered from proximal to distal, and the pretarsus is distal to the tarsus. Lengths (proximal-distal) and widths were measured as indicated by the green lines. All three of the legs have the same basic morphology. Ventral view of tarsus and pretarsus. (C) Side view of tarsus and pretarsus. (D) Scanning electron micrograph of ventral view of tarsus and pretarsus. (E) Data from multiple legs (from 10 bugs) are all shown on one leg to facilitate comparison of piercing locations. Each of the 19 red dots indicates the location where a trichome tip entered the cuticle (many are overlapping on the diagram), and each blue dot indicates an exit location. Ventral view of tarsus and pretarsus. (F) Side view of tarsus and pretarsus. (G) Scanning electron micrograph of ventral surface pierced by trichome (broken) in typical location at boundary between pretarsal claws and membrane with microtrichia on the pretarsus.

The growing resurgence of bed bugs (*Cimex lectularius* L*.*), coupled with the evolution of resistance to chemical pesticides, are stimulating the development of new non-chemical and non-toxic methods for bed bug control (Doggett et al., 2012; Hansen et al., 2011; Kells, 2006a,b; Koganemaru and Miller, 2013; Loudon, 2017; Olson et al., 2013; Potter et al., 2014; Wang and Cooper, 2011; Wang et al., 2012). In Europe, for hundreds of years, spreading leaves from bean plants around the sleeping quarters was a useful natural remedy to control bed bug populations (Potter, 2011; Richardson, 1943). Hook-like non-glandular trichomes exist on leaves from bean plants (*Phaseolus vulgaris* L*.*) (Fig. 1A), with the function of protection against aphids and leafhoppers (Johnson, 1953). Coincidentally, the same trichomes that entrap aphids and leafhoppers also entrap bed bugs despite no direct coevolutionary history between bed bugs and bean plants (Szyndler et al., 2013). The mechanism of entrapment by the hook-like plant trichomes was identified as piercing at specific locations of the tarsi and pretarsi: the undersurface of the pretarsus and between the tarsal subsegments (Szyndler et al., 2013). Once pierced, a struggling bed bug is unable to free itself from the impaling trichome. The specific mechanical barrier of the bed bug cuticle that must be overcome by the trichomes in order for piercing to occur remains uncharacterized. Identifying the underlying physical cause for the mechanical vulnerability of these specific locations on the tarsi and pretarsi can aid in the development of non-toxic, non-chemical methods of bed bug entrapment.

Whether one object will pierce another will depend on the geometry of the objects, their material and structural properties, and relative motion. The method of nanoindentation can identify the relevant material properties at a small spatial scale relevant to the piercing of bed bug tarsi and pretarsi by plant trichomes. Nanoindentation has been used to test various biological materials such as dentin (Kinney et al., 2003), bone (Huja et al., 2010), and even insect cuticle (Barbakadze et al., 2006; Broomell et al., 2008; Cribb et al., 2010; Kheireddin et al., 2012; Klocke and Schmitz, 2012; Kohane et al., 2003; Sample et al., 2015; Xiao et al., 2007); although none of the studies on insect cuticle used cuticle of legs. In addition, all studies of nanoindentation on insect cuticle except one (Kohane et al., 2003) used excised cuticle (cut from the insect). Excised cuticle dehydrates rapidly and changes in material properties over time, with the stiffness of the insect cuticle increasing as it dehydrates (Barbakadze et al., 2006; Broomell et al., 2008; Cribb et al., 2010; Kheireddin et al., 2012; Klocke and Schmitz, 2012; Xiao et al., 2007). To avoid this problem, we performed nanoindentation on live and restrained whole bed bugs at specific locations on their tarsi and pretarsi.

In order to pinpoint testing locations for nanoindentation, bed bugs entrapped by bean leaves were examined using scanning electron microscopy (SEM), allowing for more precise localization than previously presented (Szyndler et al., 2013). We then performed nanoindentation on tarsi and pretarsi in order to determine the forces required to move a sharp microscopic probe into the cuticle at two different types of locations: locations that are sometimes pierced by trichomes, and locations that are never pierced by trichomes. From these measured forces it is possible to make inferences about the material properties and piercing mechanics of the cuticle at these locations. Because the cuticle cannot be visualized *in situ* during nanoindentation, post-deformed tarsi and pretarsi were also analyzed in low vacuum SEM for residual plastic deformation. Our results show that the force needed to puncture the bed bug tarsi and pretarsi varies greatly depending on the location, allowing us to identify potential weak points along the cuticle while also beginning to quantify local mechanical properties.

## RESULTS

### Location of piercing on bed bug tarsi and pretarsi

As is true for many ‘true bugs’ (order Hemiptera), there are three tarsal subsegments (or tarsomeres) and paired pretarsal claws on each of the six legs (Woodward et al., 1970) (Fig. 1). The specific locations of piercing were imaged and mapped (as shown in Fig. 1E,F). After walking on the leaves from bean plants, each of ten bugs was pierced at least once (range 1-3 piercings/bug, average 1.9 piercings/bug). The legs were only pierced in two locations: on the membrane with microtrichia (18/19 piercings, usually near the boundary with the pretarsal claws), and through the intersegmental membrane between tarsal subsegments (1/19 piercings; similar to piercing locations reported by Szyndler et al., 2013) (Fig. 1E,F). These results were used to guide the choice of location for nanoindentation. Left and right legs were equally likely to be pierced [10 versus 9 piercings on left versus right respectively; Χ^2^ (1, *n*=19)=0.526, *P*=0.8]. In addition, the frequency of piercings at the different leg positions (forelegs, midlegs, and hindlegs) was not statistically different [Χ^2^ (2, *n*=19)=2.6316, *P*=0.27], and therefore the data are combined for all legs. The forelegs, midlegs, and hindlegs are grossly similar in morphology (Fig. 1), although the tarsal subsegments do differ slightly in length and width. For example, the third tarsal subsegment lengths and widths averaged 46 and 135 microns for the forelegs (*n*=20), 50 and 132 microns for the midlegs (*n*=16), and 51 and 151 microns for the hindlegs (*n*=15).

### Nanoindentation

**Fig. 2.**
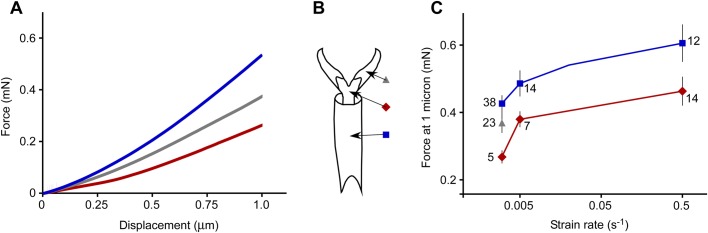
**Force required to indent to a one-micron displacement as a function of strain rate and region of the tarsus or pretarsus.** (A) Examples of nanoindenter output traces from three indents, one from each region, all at a strain rate of 0.003 s^−1^. (B) Diagram of regions on tarsus and pretarsus. (C) Forces required to move the probe of the nanoindenter 1 micron after contact with the cuticle surface (mean±1 s.e.m., sample size is indicated for each point; 113 indents illustrated overall). Red (diamonds) indicates the membrane with microtrichia, gray (triangles) indicates the pretarsal claws, and blue (squares) indicates the tarsal subsegments.

Nanoindentation was performed both at regions that are normally pierced by trichomes (the membrane with microtrichia region of the pretarsus) and in regions which are not usually pierced by trichomes (pretarsal claws and most distal tarsal subsegments) (Fig. 2B). Intersegmental membranes were too difficult to access for nanoindentation. Any indent with the nanoindenter generated an output trace of force and displacement at known time increments (Fig. 2A). The force to indent 1 micron of displacement was significantly different between membrane with microtrichia and the tarsal subsegment regions, and was significantly affected by the strain rate (Fig. 2C) [two-way ANOVA on force at 1 micron for those two regions and three strain rates: *F*(1, 86)=13.06, *P*=0.0005 for region; *F*(2, 86)=9.85, *P*=0.0001 for strain rate after removing the non-significant interaction term; *n*=90 indents total]. Specifically, the force to indent 1 micron of displacement was significantly lower for the membrane with microtrichia compared to the tarsal subsegments, and the force to indent 1 micron was greater with faster strain rates (Fig. 2C).

**Fig. 3.**
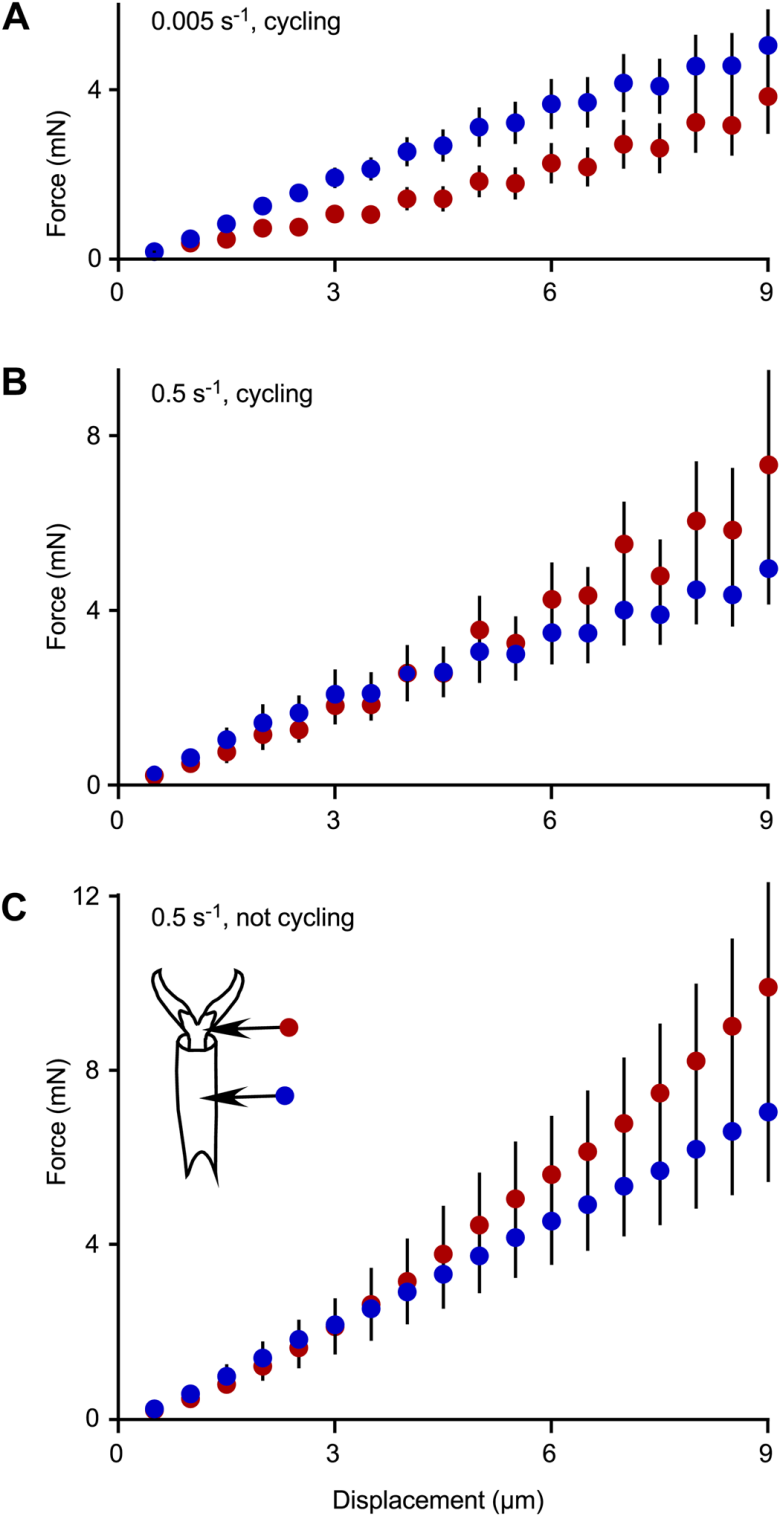
**Force to indent to different depths as a function of region of the tarsus or pretarsus.** (A) Slow strain rate of 0.005 s^−1^, cycling every micron (*n*=8 for indents in tarsal subsegments, *n*=7 for indents in membrane with microtrichia). (B) Fast strain rate of 0.5 s^−1^, cycling every micron (*n*=6 for indents in tarsal subsegments, *n*=7 for indents in membrane with microtrichia). (C) Fast strain rate of 0.5 s^−1^, not cycling (*n*=8 for indents in tarsal subsegments, *n*=6 for indents in membrane with microtrichia). Each marker indicates the mean±2 s.e.m. Red indicates the membrane with microtrichia, blue indicates the tarsal subsegments.

Indenting farther, such as a displacement of 5 microns compared to 1 micron, required a greater force for all regions (Fig. 3). These deeper indents were performed with and without cycling at one-micron increments, and at different strain rates. The increase in force with displacement was approximately linear (Fig. 3), and therefore the slope of a line fitted to the first 5 microns of displacement for any indent (forced to a zero intercept) could be used to characterize the increase in force as a single number for that indent. For the slow strain rates (Fig. 3A), the increase in force was significantly affected by region [one-way ANOVA of region on slopes: *F*(1, 13)=24.0, *P*=0.0003, *n*=15 indents]. For the fast strain rates (Fig. 3B,C), the increase in force was not significantly affected by region nor whether there was cycling or not [two-way ANOVA of region and cycling on slopes: *F*(1, 23)=0.49, *P*=0.49 for region; *P*=0.33 for cycling/not cycling after removing the non-significant interaction term, *n*=26 indents]. Comparing strain rates for the cycling indents (Fig. 3A,B), the force was significantly lower for the membrane with microtrichia than the tarsal subsegments, but was not significantly affected by strain rate [two-way ANOVA of region and strain rate on slopes: *F*(1, 25)=9.27, *P*=0.0009 for region; *F*(1, 25)=2.40, *P*=0.13 for strain rate after removing the nonsignificant interaction term, *n*=28 indents].

The age of a bed bug (days after eclosion to adult) did not significantly affect the force of indentation (at a displacement of 1 micron). An ANCOVA with strain rate as a class variable and age as a covariate was performed for each of the body regions separately (to avoid complex interaction effects); the interaction term between strain rate and age was eliminated if not significant at the 0.05 level. For the pretarsal claw region, age of the bed bugs ranged between 6 and 154 days, and did not significantly affect the force [one-way ANOVA of age on force, *F*(1, 26)=0.51, *P*=0.48, *n*=28 bugs]. For the membrane with microtrichia region, age ranged between 6 and 96 days, and did not significantly affect the force [*F*(1, 23)=0.30, *P*=0.59]. For the tarsal subsegment region, age ranged between 6 and 144 days, and did not significantly affect the force [one-way ANOVA of age on force, *F*(1, 64)=0.93, *P*=0.34, *n*=66 bugs].

### Creep measurements

**Fig. 4.**
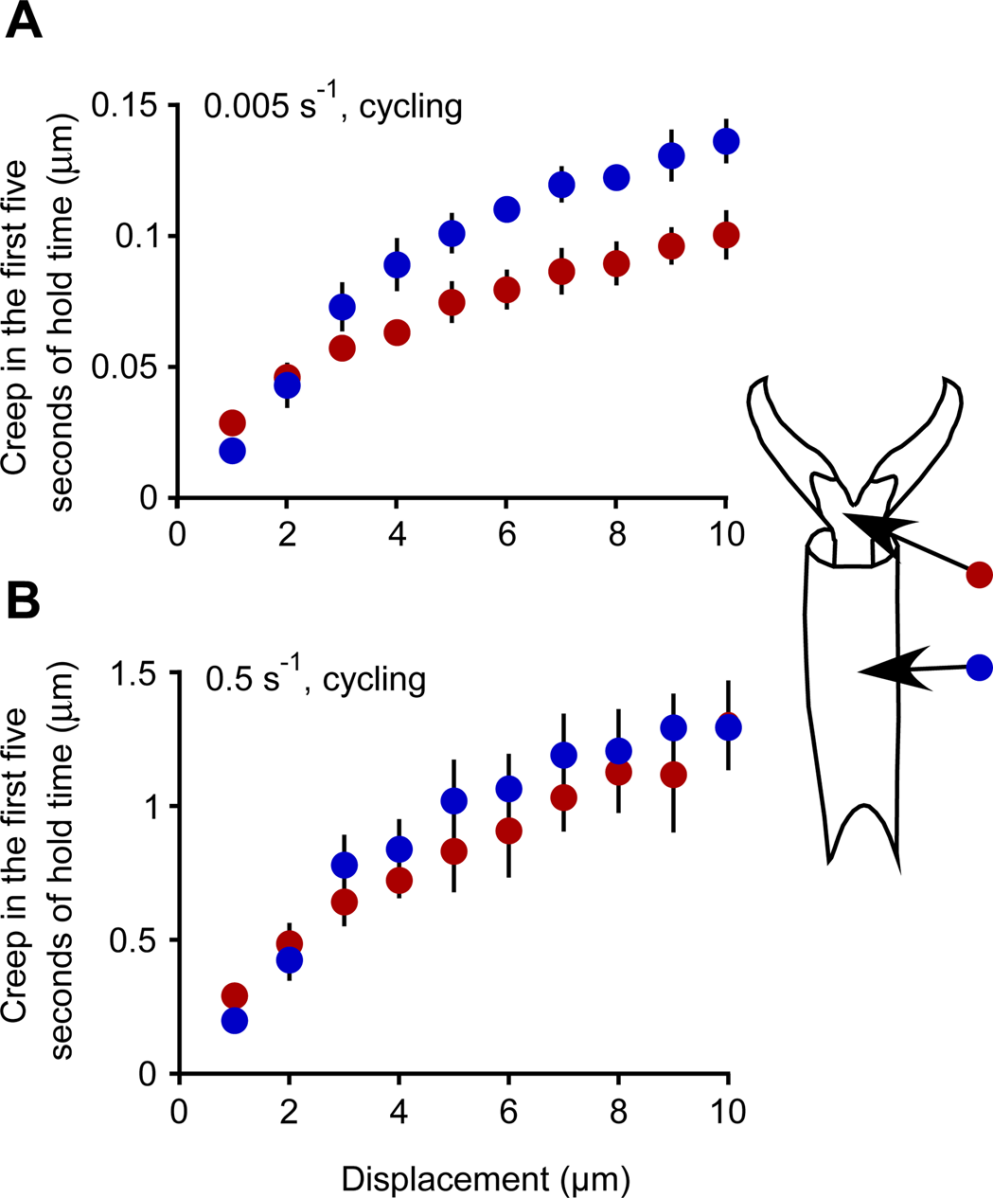
**Creep for the first 5 s of hold time as a function of strain rate and depth of indent.** (A) Slow strain rate of 0.005 s^−1^, cycling every micron (*n*=8 for indents in tarsal subsegments, *n*=7 for indents in membrane with microtrichia). (B) Fast strain rate of 0.5 s^−1^, cycling every micron (*n*=7 for indents in tarsal subsegments, *n*=6 for indents in membrane with microtrichia). Each marker indicates the mean±2 s.e.m. Red indicates the membrane with microtrichia, blue indicates the tarsal subsegments.

**Fig. 5.**
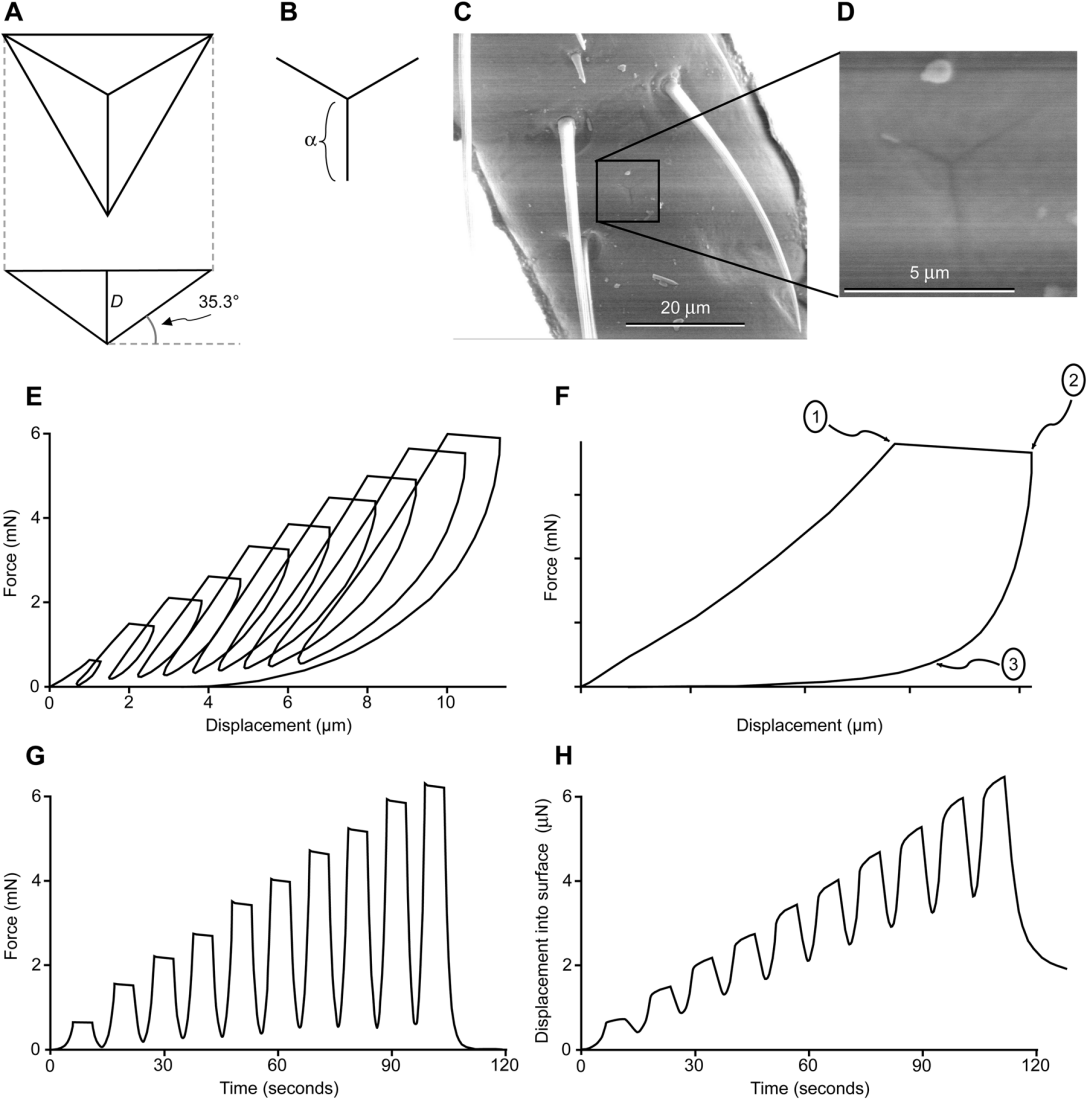
**Geometry of nanoindenter tip and output from the nanoindenter.** (A) Nanoindenter probe tip of cube corner type. (B) Shape of indent made by a cube corner tip. (C) Scanning electron micrograph of tarsal subsegment 3 (ts3) after nanoindentation. (D) Higher magnification view of indent from C. (E) Example force and displacement output during cycling. The ‘flat top’ of the loops correspond to the creep that occurs when the force is held constant (every micron of advancement for the cycling indent). (F) Marked points are used to describe how some of the calculations were made for a single cycle. Point 1 indicates the end of the loading portion of that cycle and the beginning of the hold time. Point 2 indicates the end of the hold time and the beginning of the unloading portion of the cycle. Point 3 indicates the point at which 10% of the peak load (measured at point 1) has been reached during unloading. (G) During a cycling indent, the force is cycling in time as shown for the same example trace pictured in E. After each micron of advancement, the force is held constant (for 5 s in this trace), and then reduced to 10% of its peak value before increasing again until the next micron of advancement occurs. (H) During a cycling indent, the displacement of the probe tip is cycling in time as shown for the same example trace pictured in E.

In all locations, the cuticle showed extensive creep (Fig. 4), which was measured during nanoindentation while pausing and allowing creep after each micron of advancement into the surface (as indicated in Fig. 5). After each micron of advancement, the force was held at a constant value (for 5 s for the faster strain rate and 60 s for the slower strain rate), and the cuticle continued to creep (the nanoindentor tip continued to move into the cuticle). The magnitude of creep varied with both region and strain rate (Fig. 4), and was on the order of about 25% of the displacement for fast cycling at 2 microns, and about 2.5% of the displacement for slow cycling at 2 microns (first 5 s of hold time only). For the slower strain rate (Fig. 4A), creep was greater for the membrane with microtrichia after the first micron of indentation, but was significantly lower than the cuticle of the tarsal subsegments starting after the fourth micron of indentation [a set of 10 one-way ANOVAs were performed for the 10 displacements to evaluate whether there were significant differences in creep with region; *P*<0.005 for the *F*(1, 15) values for all displacements except 2 and 3 microns; the significance level was adjusted using the Bonferroni correction as 0.05/10=0.005]. For the faster strain rate (Fig. 4B), there was no significant difference in creep with region [a set of 10 one-way ANOVAs were performed for the 10 displacements; *P*>0.005 for all *F*(1, 12) values]. For both regions (membrane with microtrichia and the tarsal subsegments), the creep was significantly greater after the faster strain (a set of 10 one-way ANOVAs were performed for each region for the 10 displacements; *P*<0.0001 for all comparisons).

Creep over the first five seconds was about 10 times as far for the faster strain rate, but the creep duration was 12 times longer for the slower strain rate (60 s versus 5 s). When the total amount of creep was compared for the two strain rates (creep in five seconds for the faster strain rate and in 60 s for the slower strain rate), the total amount of creep was only about two times as far for the faster strain rate, because the creep rate diminished over time. Therefore, providing a longer ‘hold’ time for the slower strain rate made the creep more comparable for the different strain rates (during the hold time).

### Visualization of cuticle deformation with a nanomanipulator

**Fig. 6.**
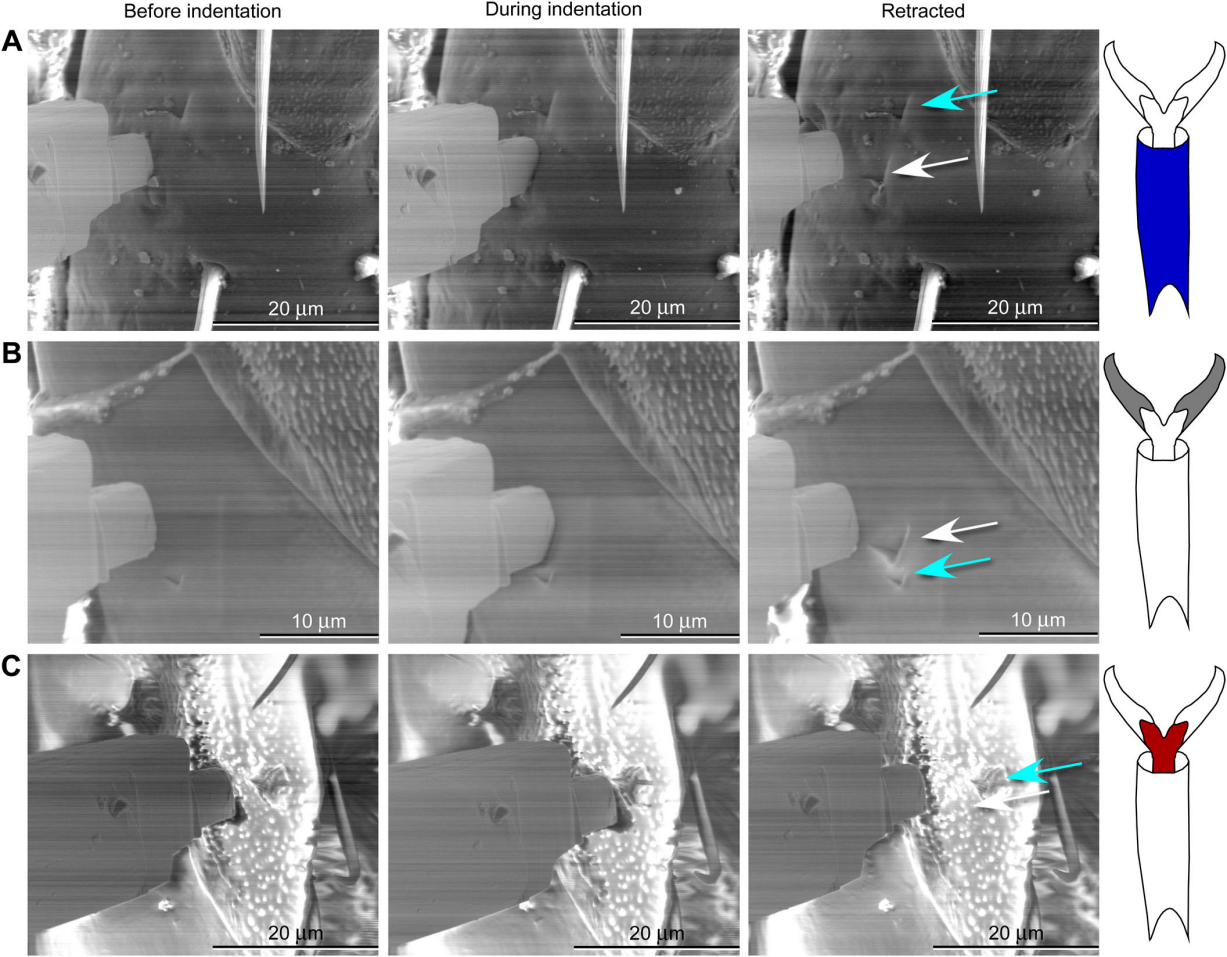
**Scanning electron micrographs of the nanomanipulator approaching the cuticle, indenting into the cuticle, and after retraction from the cuticle, leaving an indent.** In each case a previous indent is visible (blue arrow) for comparison with the current indent (white arrow). (A) Indent (3 microns) into tarsal subsegment 3 (ts3); previous indent is also 3 microns. (B) Indent (2 microns) into smooth part of claw; previous indent is 1 micron. (C) Indent (2 microns) into membrane with microtrichia region of pretarsus; previous indent is 8 microns.

Visualization of the cuticle was not possible while recording force-displacement data with the nanoindenter, and therefore, separate observations were made during low vacuum scanning electron microscopy (LV-SEM) while translating a sharp nanomanipulator probe into the cuticle (without measuring forces). Even a single micron movement of the sharp probe into the surface resulted in a visible plastic deformation of the cuticle in the smooth pretarsal claw or the tarsal subsegment (Fig. 6). In contrast, the part of the pretarsus that is not smooth (membrane with microtrichia) appears to rebound elastically without leaving any visible indentation up to 5 microns of movement, but is permanently damaged for more than 5 microns of movement (Fig. 6C). This is consistent with the results of the nanoindentation, in which small indents could be seen on the pretarsal class or tarsal subsegments (e.g. Fig. 5C,D), but not on the membrane with microtrichia. Only extensive damage (from deeper indents) was visible in the bumpy membrane with microtrichia (e.g. Fig. 6C). The tip of the nanomanipulator (Fig. 6) was a different shape (a rectangular end) from the cube corner of the nanoindentor probe (Fig. 5A), but tended to strike the surface at an angle, making a somewhat similar triangular indent (Fig. 6).

### Focused ion beam (FIB) milling to determine cuticle thickness

**Fig. 7.**
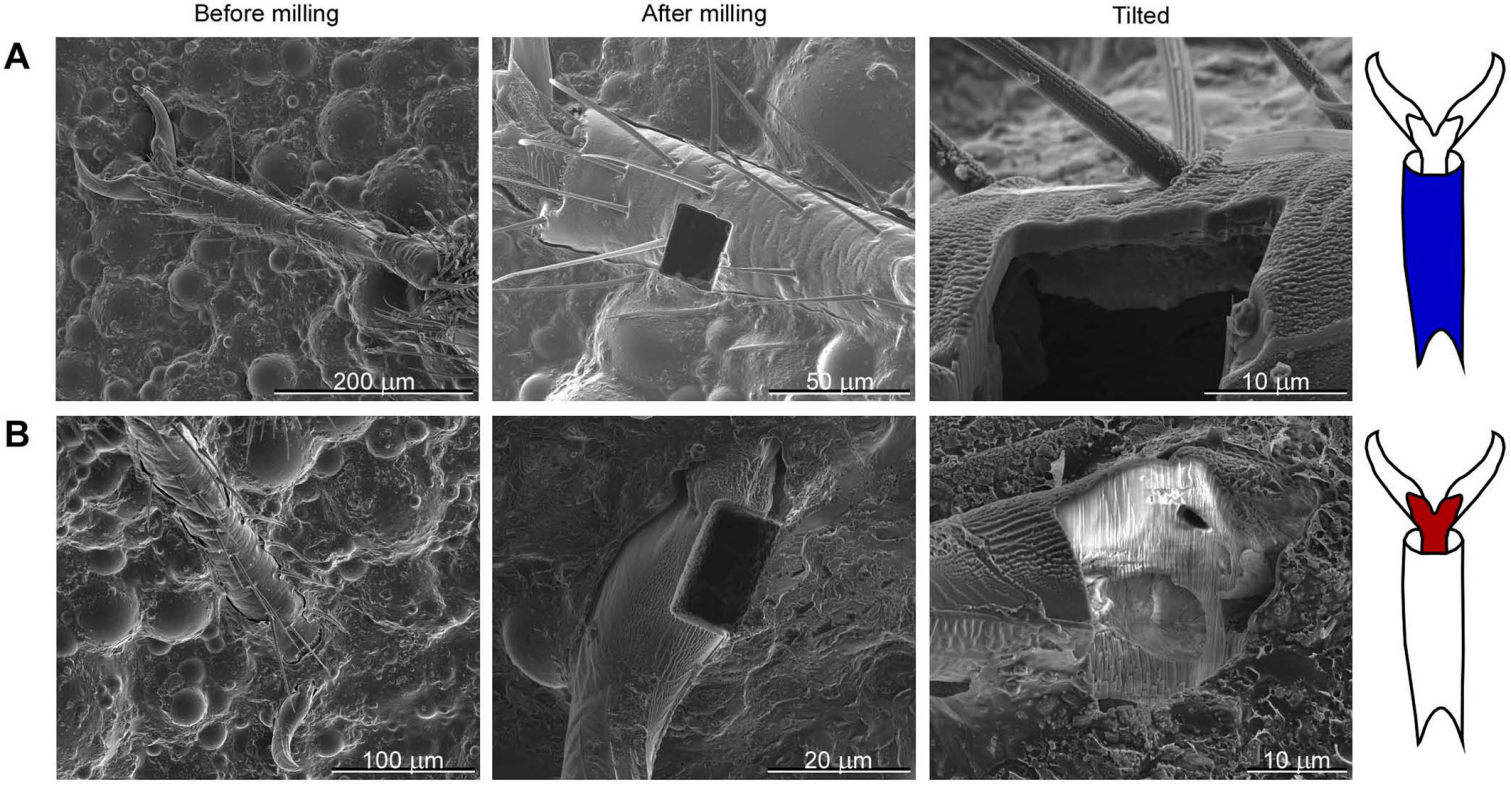
**Scanning electron micrographs demonstrate how focused ion beam milling of tarsi was used to measure cuticle thickness.** Left micrographs show tarsal structures before milling began. Middle micrographs show rectangular holes generated by milling. Right micrographs show structures tilted to view cut edges of cuticle. (A) Milling on tarsal subsegment 3 (ts3). (B) Milling of pretarsal claw and the membrane with microtrichia.

The thickness of cuticle ranged between 2-13 microns for the third tarsal subsegment (the most distal subsegment) (*n*=16 measurements on 16 legs from four bugs, mean of 7 microns) (Fig. 7). There was no significant difference in cuticle thickness of the third tarsal subsegment between bed bugs or with leg position (forelegs, midlegs or hindlegs) [two-way ANOVA of individual bed bug and leg position on cuticle thickness: *F*(3, 9)=1.63, *P*=0.25 for individual bed bug, *F*(2, 9)=0.05, *P*=0.95 for leg position]. Two additional measurements of cuticle thickness were made on the other tarsal subsegments for one leg (the more proximal subsegments), and those cuticle thicknesses (6 and 9 microns) were within the range for the most distal tarsal subsegments.

The complex shape of the pretarsal claws was reflected in the variability of the cuticle thickness. At the distal tips, the claws were completely solid, and therefore the thickness of the cuticle corresponded to the diameter of the claw at that point. Closer to the proximal end, the claws contained a complex arrangement of channels (Fig. 7B right) as also noted by Weirauch (2005) for the bug *Rhodnius prolixus*. Therefore the cuticle thickness was also highly variable for the claws (depending on the proximity of a channel), ranging from 4 to 22 microns (32 measurements on 10 legs from three bed bugs). Multiple measurements were made on single legs to capture the variability which is not accurately represented with a single measurement (Fig. 7). The membrane with microtrichia on the pretarsus, located between the pretarsal claws on the ventral surface, also showed great variation related to the presence of channels (Fig. 7B), and the cuticle thickness ranged from 2 to 33 microns (23 measurements on 11 legs from three bed bugs). Some of the thinnest cuticle was observed where the lateral edge of the pretarsal claws bordered the membrane with microtrichia (the usual location of piercing, Fig. 1E): a cuticle thickness of 3 microns was made at this boundary.

### Reduced elastic modulus measurements

**Fig. 8.**
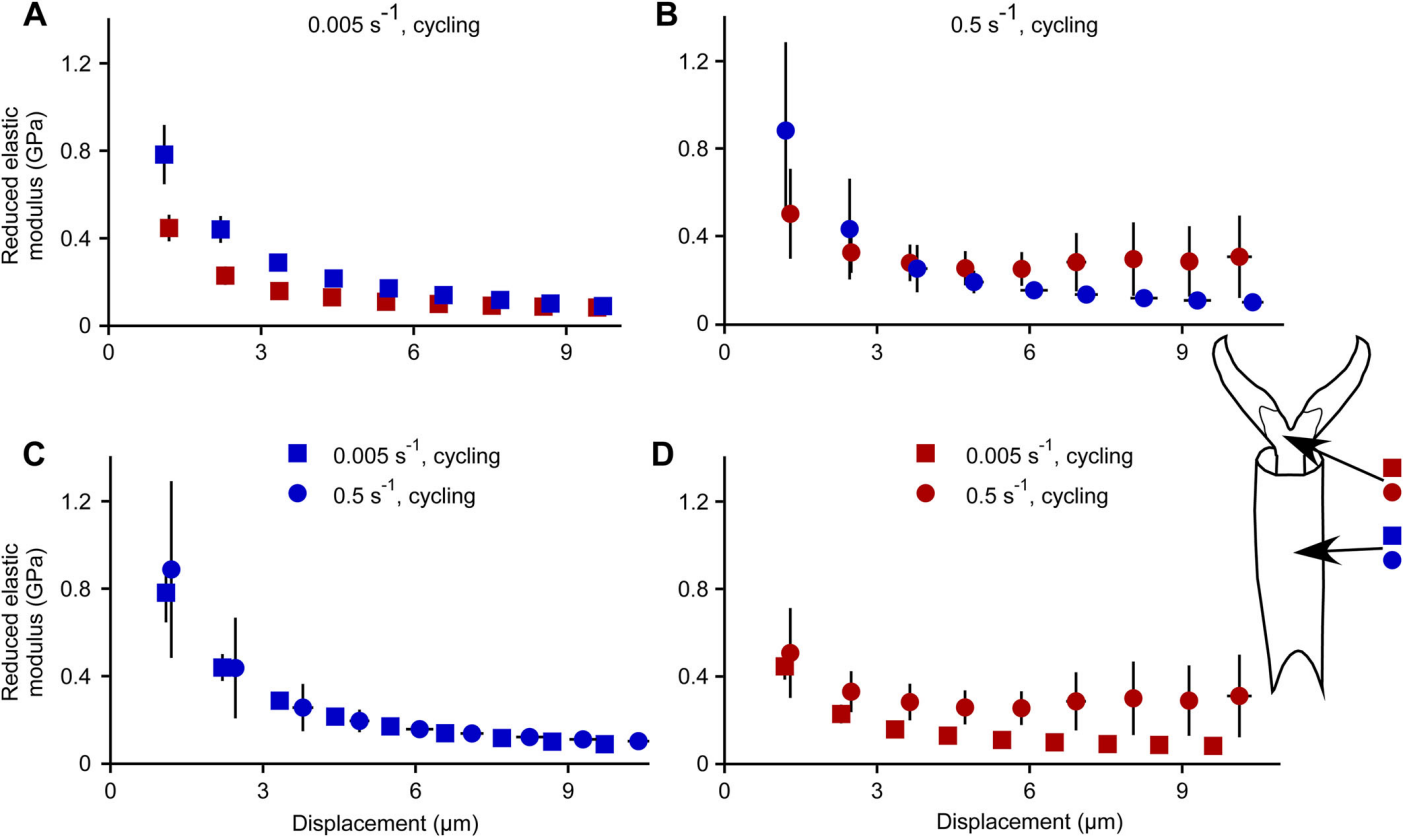
**Reduced elastic modulus as a function of displacement during nanoindentation.** (A) Slow strain rate only, comparing the two regions. (B) Fast strain rate only, comparing the two regions. (C) Tarsal subsegment 3 (ts3) only, comparing the two strain rates. (D) Membrane with microtrichia only, comparing the two strain rates. Each marker indicates the mean±2 s.e.m. for both *y* and *x*. Sample sizes: for slow strain rates, *n*=8 for indents in tarsal subsegments, *n*=7 for indents in membrane with microtrichia; for fast strain rates, *n*=7 for indents in tarsal subsegments, *n*=6 for indents in membrane with microtrichia. Red indicates the membrane with microtrichia, blue indicates the tarsal subsegments.

Reduced elastic modulus was calculated from the nanoindentation data during unloading after each hold time for each micron cycle. There are estimates of reduced elastic modulus at approximately one-micron increments; however, these are not exactly one-micron increments because of creep, and reduced elastic modulus is reported based on the displacement after creep has occurred (Fig. 8). For the slow strain rate (Fig. 8A), reduced elastic modulus was significantly different for the regions for the first 4 microns only, with a larger modulus in the tarsal subsegment compared to the membrane with microtrichia (separate one-way ANOVAs for each displacement cycle; *P*<0.005 for region for first four cycles, *P*>0.005 for region for the remaining five cycles; d.f.=1; the Bonferroni correction for the significance level is 0.05/9=0.0055). For the fast strain rate (Fig. 8B), the reduced elastic modulus was not significantly different for the two regions for any of the displacements (separate one-way ANOVAs for each displacement cycle; *P*>0.005 for all; d.f.=1). Reduced elastic modulus was not affected by strain rate for cuticle of the tarsal subsegments (Fig. 8C) (separate one-way ANOVAs for each displacement cycle; *P*>0.005 for all cycles; d.f.=1). For the membrane with microtrichia on the pretarsal claws, reduced elastic modulus was affected by strain rate after the fifth micron (Fig. 8D) (separate one-way ANOVAs for each displacement cycle; *P*>0.005 for strain rate for all cycles except the fifth, *P*<0.005 for strain rate for the fifth cycle; d.f.=1).

The elastic modulus, *E*_cuticle_, can be calculated from the reduced elastic modulus, *E_r_* (Eqn 10), if the Poisson's ratio for cuticle is known, in addition to the material properties of the nanoindenter probe tip. The probe tip is made of diamond. The relevant properties for diamond are *v*_diamond_=0.07 (dimensionless) and *E*_diamond_ =1143 GPa (Klein, 1992). The Poisson's ratio for insect cuticle has been reported as varying between 0.02 and 0.1 [in the plane of the cuticle, locust intersegmental membrane cuticle on the ovipositor of adult female locusts (Vincent, 1990); different numbers graphed in fig. 4.12 of the same reference are assumed to be in error]. Substituting in these values for diamond and cuticle into Eqn 10, it is clear that the term for the indenter is negligible, leaving the simplified relationship:
(1)
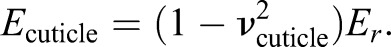
If the Poisson's ratio for the bed bug cuticle is ≤0.1, this would mean that *E*_cuticle_ would be within 1% of *E_r_* (Eqn 1), and therefore is a close approximation. The reduced elastic modulus is often reported because of the lack of information on the Poisson's ratio for insect cuticle (Muller et al., 2008).

## DISCUSSION

As an insect or other arthropod walks, its tarsi and pretarsi, which are in contact with the surface, are potentially vulnerable to damage or entrapment. For example, aphids and leafhoppers moving on the surface of a bean plant are entrapped by hook-like trichomes which pierce the tarsi or pretarsi and hinder their mobility (Johnson, 1953). We have identified the magnitude of the force required to indent the cuticle in various regions of bed bug tarsi and pretarsi at various depths by a sharp probe. The magnitude of the force to indent one micron into the cuticle was approximately 0.5 mN (averaging over all regions and strain rates). A larger force is required to indent farther into the cuticle for all regions. The region of the pretarsus that is typically pierced, the membrane with microtrichia, differed in several respects from the other areas of cuticle on the tarsus, but only for the first micron of indentation. The membrane with microtrichia was easier to indent (about 20-30% less force for a depth of 1 micron, Fig. 2), displayed more creep (Fig. 4), and had a lower reduced elastic modulus (slow strain rates only, Fig. 8) than the tarsal subsegments (Fig. 8). For deeper indentations (>1 micron), these differences between regions either disappeared or reversed (Figs 3, 4 and 8). The significance of these differences for shallow indents may lie in facilitating the start of a puncture by a sharp trichome. In addition, the membrane with microtrichia behaves differently from the other regions tested. When visualized in low vacuum SEM while probing with the sharp nanomanipulator, the membrane with microtrichia did not form the characteristic indentation shape (dent) as seen in other areas of the cuticle, but simply rebounded without visible damage to a depth of up to 5 microns when the nanomanipulator probe was retracted. All of these mechanical attributes, in addition to the presence of the natural infoldings in the cuticle of this area, may make the membrane with microtrichia area of the pretarsus particularly vulnerable to piercing.

A larger force is needed to indent farther into the cuticle (Fig. 3). Therefore, it might be expected that a larger force would be required to puncture thicker cuticle. The cuticle thickness was highly variable for all areas examined, although the variability showed a different pattern for the different regions. For the pretarsal region (pretarsal claws and membrane with microtrichia), when the cut surface of the cuticle was examined after milling, the cuticle thickness varied on a single specimen over a distance of a few microns because of the channels that infiltrated the pretarsus in a complex manner (measuring cuticle thickness from the outside to a channel, Fig. 7B). The piercing by trichomes was usually close to the boundary between the membrane with microtrichia and a pretarsal claw, and the cuticle was often observed to be particularly thin at this boundary area; although the abrupt changes of cuticle thickness in any direction made it challenging to measure consistently. In addition, the infolding of the cuticle at that boundary could help to secure the tip of a sharp trichome, analogous to a ‘starter hole’ guiding a hammered nail. A more thorough three-dimensional analysis of the cuticle of the pretarsal region would be needed to provide a systematic and comprehensive description of its complex geometry. Cuticle thickness was less variable on a single specimen for an individual tarsal subsegment. For the tarsal subsegments most of the varability in cuticle thickness was seen between individuals, not within a single tarsal segment, in which the cuticle thickness did not vary appreciably over tens of microns (Fig. 7A). Although force data were recorded to a probe movement of 9 microns, whether or not a sharp object will penetrate and possibly ultimately pierce the cuticle may be largely determined by the mechanical interaction that occurs in the first few microns.

The thickness of the cuticle might have a complex relationship with the force needed to pierce it. For example, an area with thin cuticle might be more likely to simply bend macroscopically and deform without being pierced, while an area with thicker cuticle might be less likely to bend and therefore be more likely to be pierced. That is, stiff structures are easier to break than deformable ones (Vogel, 1986). The boundary between the membrane with microtrichia and the pretarsal claw has a complex geometry that might explain why this area is particularly vulnerable to piercing: the boundary is composed of a particularly thin area of cuticle surrounded by thicker areas of cuticle. Thus, in addition to the infolding that might help to secure a sharp point, the thinner area of cuticle might be ‘held in place’ by the surrounding thicker cuticle and therefore unable to simply deform out of the way, and being thinner, is easier to pierce.

We did not observe an abrupt discontinuity, or sudden change in the force recording, as the sharp indenter probe completely penetrated the cuticle. Therefore, we are unable to identify the exact displacement at which the cuticle was pierced completely by the probe, even though the cuticle would eventually become punctured with sufficient depth. We know that the cuticle was usually pierced after a 10 micron displacement for both the tarsal subsegments and the membrane with microtrichia, because we were able to visualize the holes during later examination of the same bugs in LV-SEM. The deformations that we generated with our sharp probe were localized to the immediate area of the sharp probe. The nanoindenter probe tip that we used has a shape of a cube corner with a sharp tip, and shares that important similarity with real trichomes which have a sharp but conical tip (round in cross-section). The nanomanipulator probe tip was not as sharp as the nanoindenter tip, and had a slightly different shape of a rectangular cross-section, but also made a triangular indent into the cuticle surface.

The time-dependent properties of cuticle suggest that more force would be required to pierce the tarsi if the tarsi were moved more quickly during locomotion. Insects (including bed bugs) tend to cycle their legs through tripodal movements at a high frequency (bed bugs cycle through all six legs at ∼8 times/sec; Szyndler et al., 2013), which means that their tarsi are being impaled within a fraction of second. This is even faster than our fastest experimental strain rate, but we were constrained by machine capabilities. Evidently bed bugs are generating sufficient force when moving their feet to self-impale, but this force has not yet been measured. We know of no documented cases in which insects move particularly slowly when around trichomes, although careful placement of tarsi to avoid trichomes has been documented in other insects walking on plant surfaces (Voigt et al., 2007; Voigt and Gorb, 2010). In other piercing contexts, it suggests that piercing of cuticle by ovipositors of parasitoid wasps or piercing of cuticle by the aedeagus during traumatic insemination in bed bugs could require less force if the piercing structure is moved more slowly. Mechanical creep reduces the force necessary to pierce an object because a sharp object pressed into the cuticle will continue to sink into the cuticle. The time course of mechanical creep of insect cuticle can be very long: the cuticle of fifth instar *Rhodnius* bugs was still exhibiting creep 8 h after a constant load was applied (Reynolds, 1975).

The original application for nanoindentation was to test homogenous, flat materials such as thin films and crystalline structures (Oliver and Pharr, 1992). Bed bug cuticle on live, restrained whole bed bugs is neither homogenous nor flat. Therefore, the estimates of material properties of the cuticle, such as the elastic modulus, would be expected to show variability, which was observed. However, even with this variability (also documented by Sample et al., 2015, for insect wing cuticle), clear differences in mechanical properties of the different parts of the tarsus and pretarsus were documented for the small indents of 1 micron. In addition, the forces measured using the sharp nanoindenter tip (‘cube corner’ geometry) may be used to estimate the forces that would be required for a plant trichome to pierce the cuticle.

The identification of the forces required to pierce bed bug cuticle and the mechanical properties of the cuticle should help to inform development of synthetic materials intended to functionally mimic the bed bug entrapping capabilities of leaves from bean plants. In addition, this mechanical characterization will help us understand the coevolutionary arms race between trichome-armed plants and insects.

## MATERIALS AND METHODS

### Experimental insects and plants

All bed bugs used in experiments were adult males (*Cimex lectularius*). Bugs were of the ‘Fort Dixon’ strain and were supplied by Dr K. Haynes of the University of Kentucky, USA. Bugs were not fed after eclosion to adults. Measurements were made on bugs 6–154 days after adult eclosion (mode was 18 days, mean was 53 days).

Kidney bean plants (*Phaseolus vulgaris*) were grown in the University of California, Irvine, USA greenhouse from seed (Johnny's Seeds, Winslow, ME, USA; Product 2554). Individual leaves used for bed bug entrapment were trifoliate and node ≥1 because these are the leaves that are most effective in capturing bed bugs (Szyndler et al., 2013). All leaves used were <90 mm in length (from tip of leaf to petiole attachment).

### Scanning electron microscope (SEM) imaging

Low-vacuum scanning electron microscope (LV-SEM) imaging was performed on a Quanta 3D FEG Dual Beam SEM (FEI, Hillsboro, OR, USA) at 0.6 mbar and 5 kV. Bed bugs examined in LV-SEM were glued onto SEM stubs with an acrylic mounting system (VariDur high performance mounting system; Buehler, Lake Bluff, IL, USA) to restrict their movement. Bugs were alive during and after imaging and exposure to the vacuum.

### Determining specific locations of piercing

Leaves were removed from the plants, and placed with their abaxial side (undersurface) facing upward. Bed bugs (*n*=10) were placed individually on the leaves and were allowed to walk until they became entrapped by the hook-like trichomes on the leaves. These pierced bed bugs were removed from these leaves and placed dorsal surface down on carbon tape on SEM stubs. The bug tarsi were viewed in LV-SEM and examined for evidence of piercing by bean plant trichomes.

### Anatomy of tarsal subsegments

In order to evaluate gross differences in morphology in the different legs, lengths and widths (diameters) of tarsal subsegments were measured from scanning electron micrographs using Canvas 15 (ACD Systems, Seattle, WA, USA) (see Fig. 1B,C,D) for 23 bugs. LV-SEM images were used to determine the length and width of the tarsal subsegments for the six different legs (*n*=120 legs from 23 different bugs; 1-6 legs were used per bug; 3 bugs had all 6 legs measured).

### Nanoindentation

For nanoindentation, live bed bugs were mounted on SEM stubs with an acrylic mounting system as described above. Live bugs were mounted on their backs, with their dorsal surface in contact with the SEM stub so that the ventral side of the bug was accessible. The tarsi, abdomen, thorax, and head were all firmly fixed in place to minimize movement during experimentation, leaving parts of the tarsi and pretarsi exposed for nanoindentation. Bugs were still alive after nanoindentation. If there was any evidence of tarsal movement, those data were not used. A total of 123 indents on 67 different legs of 47 different bugs were analyzed. After nanoindentation, bugs were moved to the scanning electron microscope to visualize the indents using LV-SEM as described above (Fig. 5).

Mechanical testing was performed with an Agilent G200 nanoindenter (Agilent, Santa Clara, CA, USA) fitted with a diamond cube-corner tip with testing performed in DCM mode at room temperature. The cube-corner probe exhibits the geometry of the corner of a cube, allowing for a relatively sharp tip that when pressed into the surface makes a characteristic three-sided indent (Fig. 5). For a cube corner (‘trirectangular tetrahedron’), the relationship between the area of the triangular base (*A_b_*) (the projected equilateral triangle viewed in Fig. 5A) and the altitude of the tetrahedron (the depth of indentation, *D*) is:
(2)
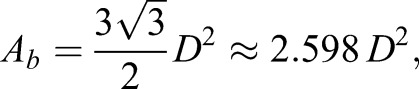
and the relationship between the projected length of any of the three ‘legs’ (α, seen in Fig. 5B) and the depth of indentation is:
(3)
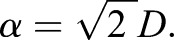


Four different indentation strain rates were used: 0.003, 0.005, 0.02, and 0.5 s^−1^. These are ‘true’ strain rates (instantaneous rates of increase in length), not ‘engineering’ strain rates (rates of increase in length compared to starting length) (Lucas and Oliver, 1999; Vogel, 2013). Strain rates, *ɛ˙_i_*, were evaluated directly from the raw displacement and time data by dividing the instantaneous rate of displacement by the average displacement during that same time period:
(4)
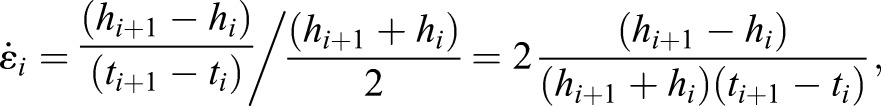
where *h_i_* is the displacement corresponding to time *t_i_* (therefore, *ɛ˙_i_* is a forward estimate). The instrument was used in strain rate controlled mode, with force and displacement recorded continuously.

In order to uncover any inhomogeneous material properties as a function of tip displacement, some of the indentation experiments were performed with cycling. For each cycling test the probe penetrated in micron increments, pausing with a constant force at each depth for a set hold time (60 s for a 0.005 s^−1^ strain rate and 5 s for a 0.5 s^−1^ strain rate) in order to observe any time-dependent material response. Creep was measured as the continued displacement into the cuticle during the force hold for the set time period. Creep during the hold time is clearly taking place, as can be seen in Fig. 5. Mechanical properties were calculated from unloading segments performed after each holding segment before continuing to the next depth. Unloading consisted of retracting the probe to 10% of its peak load value while still maintaining contact with the surface.

Reduced elastic modulus was calculated from the nanoindenter output using the Oliver-Pharr method (Oliver and Pharr, 1992) with the recommended adjustments for viscoelastic materials such as polymers (Kinney et al., 2003; Ngan et al., 2005) due to the creep strain that was observed. The average displacement rate during hold time, 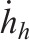, is:
(5)
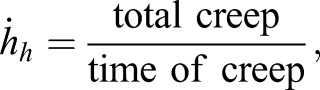
where the ‘total creep’ during the hold time is Δ*x* between points 1 and 2 in Fig. 5B, and the time of creep is 5 s for the fast strain rate (0.5 s^−1^) and 60 s for the slow strain rate (0.005 s^−1^). A longer time for creep was provided for the slower strain rates to make the total creep more similar between the different strain rates (the rate of creep during the hold time was slower for the slower strain rates). The average unloading rate during the first 90% of unloading, 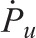, was calculated by:
(6)
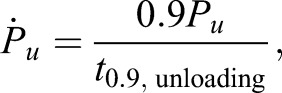
where *P_u_* is the force at the beginning of unloading (the *y* value of point 2 on Fig. 5B) and *t*_0.9, unloading_ is the time difference from the beginning of unloading until the load has reached 10% of its initial unloading value (the time difference between points 2 and 3). This approach is comparable to Kinney et al. (2003), who estimated 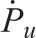 from the midpoint of the unloading curve (force versus time). The rate of change of force during unloading is fairly linear (Fig. 5C), and therefore Eqn 6 should provide similar results to the approach in Kinney et al. (2003). The apparent stiffness, *S*, is output by the nanoindenter software, and is the slope of the linear fit to the initial 50% of the unloading region of the curve. The creep-corrected contact stiffness, *S_e_*, is calculated as (Ngan et al., 2005):
(7)
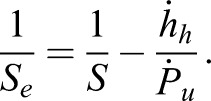
The reduced elastic modulus, *E_r_*, is calculated as:
(8)
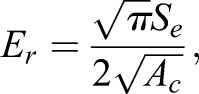
where *A_c_* is the ‘contact area’ (projected area, as viewed in Fig. 5). *A_c_* is calculated from the basic geometry of a tetrahedron (Eqn 2) substituting *h_c_* in for the altitude, *D*, where the contact depth, *h_c_*, is calculated as:
(9)
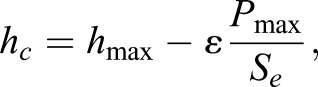
where *h*_max_ is the maximal displacement during the cycle (*x* value of point 2 in Fig. 5B) and ε is a number dependent on probe tip geometry, which is 0.75 for ‘conical’ indenter tip shapes (i.e. both cube corner and Berkovich tip shapes; Oliver and Pharr, 1992; Pharr, 1998).

Calculation of the elastic modulus, *E*_cuticle_, from the reduced elastic modulus, *E_r_*, requires knowing the Poisson's ratio, *ν*, for the cuticle and the indenter, as well as the elastic modulus of the indenter tip *E*_indenter_ (Oliver and Pharr, 1992):
(10)
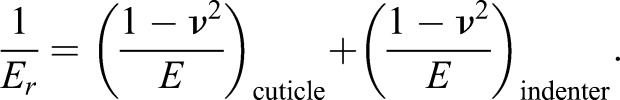


### Visualization of cuticle deformation with a nanomanipulator

A nanomanipulator (Omniprobe 200, Oxford Instruments, Oxford, UK) was used while bugs were in being viewed in LV-SEM (FEI Quanta 3D FEG Dual Beam SEM) to visualize the events that occur when deforming bed bug cuticle with a sharp probe (such as during nanoindentation, which cannot be observed directly). To facilitate comparison with the nanoindenter data, the nanomanipulator deformations were also made on the third tarsal subsegment and two regions of the pretarsus (the pretarsal claws and the membrane with microtrichia). The shape of the nanomanipulator probe tip was similar to the nanoindentation probe, with a sharp corner, and is solid tungsten (*E* for tungsten is 340-380 GPa; Gere and Timoshenko, 1997).

The nanomanipulator probe was moved to a position where it lightly made contact with the surface of the bed bug cuticle without deforming the cuticle. The nanomanipulator probe was then moved into the cuticle at a speed of 0.5 microns s^−1^ for depth in the range 1−8 microns. Displacement into the surface of the cuticle was verified by removing the probe in one-micron increments until the nanomanipulator probe had clearly lost contact with the surface.

Images were taken before insertion of the nanomanipulator probe, during the maximum translation of the nanomanipulator probe, and after removal of the nanomanipulator probe in order to categorize the type of deformation (e.g. plastic versus elastic) (Fig. 6). A total of five legs from five bugs (one leg from each bug, right or left, midleg or hindleg) were each probed at least one time on each of the hree regions per leg: pretarsal claws, membrane with microtrichia on the pretarsus, and tarsal subsegments. A total of 30 insertions ranging from 1−10 microns in depth were made.

### Focused ion beam (FIB) milling to determine cuticle thickness

FIB was performed using the FEI Quanta 3D FEG Dual Beam SEM (FEI, Hillsboro, OR, USA) at 1.94×10^−5^ mbar, 16-30 kV and 15 nA using a beam of gallium ions for milling. Specimens were air-dried or critical-point dried and sputter-coated with Ir (5-7 nm deep) before milling with FIB. At least three tarsi on each of three bugs were milled in the claw and tarsal subsegments to allow estimation of cuticle thickness from SEM images recorded while milling. The images (1024×943 pixels, tiff format) were imported into Canvas 15 (ACDSee, Seattle, WA, USA), the scale determined by the scale bar on the image, and the cuticle thickness measured from the image, rounded to the nearest tenth of a micron. The cut cuticle was viewed at a known angle relative to the milling plane; this angle was typically between 10 and 55°, because it was not possible to view it at a 90° angle normal to the milling plane. The corrected cuticle thickness was calculated by dividing the thickness measured from image by the sine of the angle relative to milling plane to correct for the foreshortening caused by the viewing angle, and rounded to the nearest micron.

### Statistics

Statistical tests were performed using SAS 9.4 (SAS, Cary, NC, USA). The Proc GLM (General Linear Models) procedure was used for two-way ANOVAs, the interaction terms were included except when noted otherwise, and Type III results are reported. In the Type III model, the order of the parameters does not matter because each effect is adjusted for all other effects (SAS Institute Inc., 2008). The Proc GLM was also used for one-way ANCOVAs. The Proc FREQ procedure was used for chi-square testing. For multiple comparisons, the Bonferroni correction was applied to the significance level by dividing by the number of comparisons (e.g. Sokal and Rohlf, 1995; Whitlock and Schluter, 2015).
